# The influence of ophthalmological diseases on the vision quality of famous painters

**DOI:** 10.22336/rjo.2021.67

**Published:** 2021

**Authors:** Camelia Margareta Bogdănici, Irina Andreea Niagu, Daniela Gabriela Andronic

**Affiliations:** * Discipline of Ophthalmology, “Grigore T. Popa” University of Medicine and Pharmacy, Iași, Romania

**Keywords:** ophthalmology, art, paintings

## Abstract

This article represents an engagement between ophthalmology, culture, art, and knowledge. The paper reviews numerous ophthalmic pathologies that affected some of the most famous artists from all the time. The style of a painting can be created on purpose by the artist, but it can also be affected by the visual acuity. The most significant ocular diseases that might affect visual acuity and style of painting are represented by strabismus, refractive errors, cataract, retinal diseases, color vision deficiency and ocular trauma. During the time, various styles of paintings could be encountered and, very often, we wondered whether the style is influenced by the visual acuity of the artists or visual acuity does not affect at all the styles. The purpose of this study was to prove how ocular pathologies might have affected art creation during the past centuries.

## Introduction

Sight represents an indispensable tool for creating works of art. Without sight, artists cannot guide their brush movements over the canvas and cannot observe the color and shape of their art work.

There is a long history of scientists and clinicians claiming that certain artists were affected by visual pathologies, based on the signs in their works. For example, some have argued that the leaders of the Impressionist movement had myopia and their blurred vision at distance, when not wearing glasses, might explain their impetuous style.

The evidence supporting such disorders and their influence on works of art is often speculative and is hampered by a lack of clinical records to support the diagnosis. A special challenge in verifying these speculations is that artists are, of course, free to represent the world in any way they prefer [**1**].

## Method

We searched PubMed and Scopus databases using the following keywords: ophthalmology, art, paintings, to find relevant articles on the selected topic. The most important articles of art have been analyzed to identify famous artists who suffered from various ocular diseases. We identified artists whose ophthalmological pathologies were described in detail and for whom adequate information could be obtained in order to allow the investigation of their ocular diseases and the effects on each artist’s work. Specific illustrations to the work of each artist were obtained. Access to a lot of art pieces is extremely difficult, because they are owned by various private collectors.

## Results and discussion

*Strabismus*

**Fig. 1:**
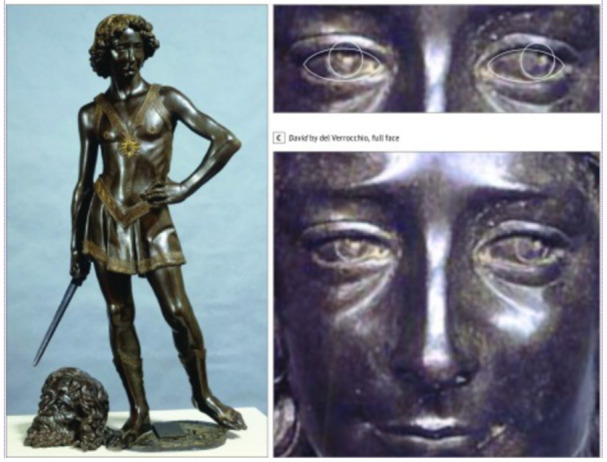
David by Andrea del Verrocchio [**2**]

Leonardo da Vinci was born on April 15th 1452 in Italy. He had many talents and research interests, including painting, drawing, sculpture and more. Only a part of his paintings survived, Mona Lisa being the most famous opera. After examining six of his works in three different techniques (drawing, painting and sculpture), the researchers noticed that the subject’s eyes were pointing outward in each of his works, which is suggestive for exotropia. Bronze sculpture of David by Andrea del Verrocchio is considered a description of young Leonardo da Vinci and it demonstrates the artist’s exotropia (**Fig. 1**) [**2**].

Guercino was an artist from Italy from the 1600s who used the baroque art style in more than 200 paintings that he created during his life. It is said that he suddenly developed strabismus one night after waking up due to an extremely loud and suspect noise that led to the appearance of esotropia in the right eye. Many of his works feature characters with strange or unusual facial features, aspect which is more evident in his caricature drawings [**3**].

**Fig. 2:**
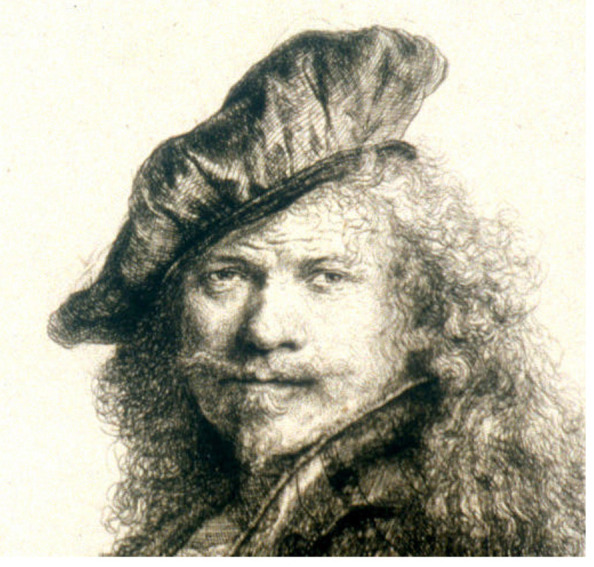
Self Portrait - Rembrandt, 1639 [**4**]

Rembrandt van Rijn is another famous artist from the 1600s. He was from Netherlands and is considered one of the greatest artists in the history of art, being trained as a sketcher, printer, and painter. Many of his self-portraits show the eyes pointing outwards, which is suggestive for exotropia. In 1639, Rembrandt painted a self-portrait leaning on a stone wall, which shows his exotropia at the left eye (**Fig. 2**). Artist’s monocular vision has been often seen as an advantage, as it has been considered that he could easily observe the subject’s details that his colleagues with binocular vision might not observe [**4**].

Pablo Picasso was born on October 25th 1881 and despite the fact he was born in Spain, he lived in France for much of his adult life. It is believed that he contributed to the creation of the Cubist movement and he worked in a variety of fields, including painting, sculpture, printing, ceramics and more. Some researchers consider that his interest in Cubist painting stems from his lack of perception of depth, which is suggestive for the absence of stereopsis, the third stage of binocular vision. Picasso preferred to show off his shading skills whenever possible in his 2 D works. Some paintings suggest that Picasso may have suffered from migraine. This condition is associated with the illusory division of images, which could inspire his characteristic way of painting the women’s faces. However, there is no official evidence that Picasso suffered from this condition [**5**].

*Retinal diseases*

Edgar Degas was born on July 19th 1834 in France. He is considered one of the creators of the French Impressionist movement, having more than 50% of his artistic pieces related to dance. The ophthalmological symptoms were noticed by the artist for the first time in 1870 while visiting his family, when he realized that he had problems with painting in bright sunlight. The artist’s central vision was impaired, making many of his following paintings seem blurry. His sister had a similar eye condition and completely lost her visual acuity at the age of 30, and, because of this, it is believed that the ocular condition was genetic [**6**,**7**].

**Fig. 3:**
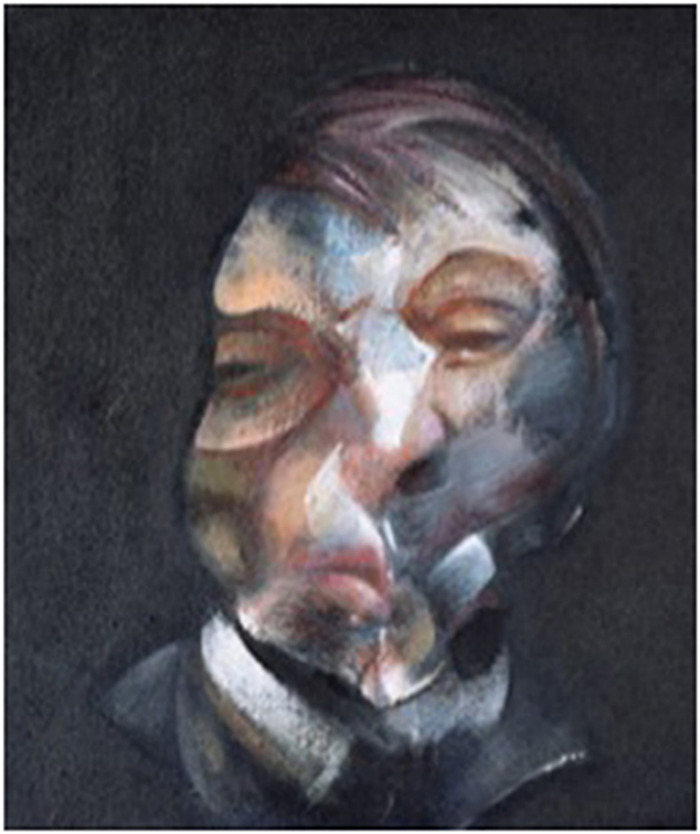
*Self-portrait* – Francis Bacon, 1971 [**8**]

Francis Bacon was a British artist of Irish descent from the 1900s who was known for his unreal and terrifying work. In many interviews and discussions with art critics, he described how images seemed to be continuously changing, almost like an optical illusion. This fact is reflected in his work, which presents strongly distorted images with anomalies of the faces. The self-portrait painted by the artist in 1971 illustrates these anomalies (**Fig. 3**). This is consistent with metamorphopsia that affects a person’s ability to perceive the shape of objects [**8**].

Georgia O’Keeffe was an artist from the 1900s and she was from United States of America. She is known for her flower paintings, New York skyscrapers and New Mexico landscapes. In 1964, she began experiencing symptoms of age-related macular degeneration for the first time. She described the condition as a cloud that comes into her eyes. Moreover, Georgia O’Keeffe suffered from retinal vein occlusion in the left eye in 1971 [**1**].

Mary Cassatt, born on May 22nd 1844 in the United States of America, was a member of the Impressionist movement. Many of her paintings illustrate different aspects from women’s live, with a special focus on mothers with their children. She began to lose her visual acuity at the age of 56, describing it as becoming lower and lower. The artist was diagnosed with cataract and diabetic retinopathy 10 years later. She went from using oil paintings to pastels, favoring large canvases instead of smaller ones, and began to draw bolder lines in her work instead of complicated details. She also used fewer colors due to the deterioration of her color vision [**9**].

*Refractive errors*

Auguste Renoir was born on February 25th 1841 in France. He was a member of the Impressionist movement and he was famous for his portraits. It is also known that he had myopia. Despite this fact, he never wore glasses and considered his ophthalmological condition to be an advantage in landscape painting because his works looked perfectly blurred [**10**].

**Fig. 4:**
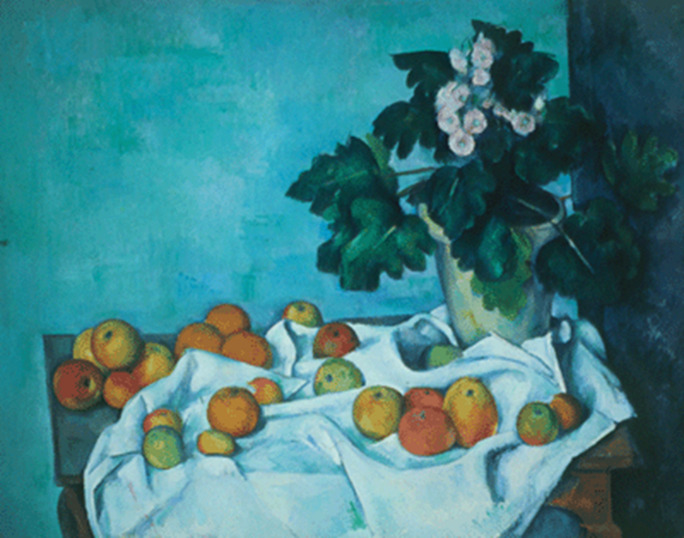
*Still life: apples and a pot of primroses* - Paul Cézanne, 1890 [**10**]

**Fig. 5:**
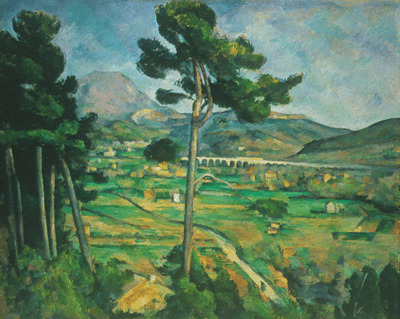
*Mont Sainte-Victoire and the Viaduct of the Arc River Valley* - Paul Cézanne, 1882-1885 [**10**]

Paul Cézanne was another artist who had myopia and he suffered from diabetes, too. The images painted at near distances are transposed into clear, distinct works, while his landscape paintings that highlight images observed at distance, are less clear and more distorted. “Mont Sainte-Victoire and the Viaduct of the Arc River Valley” realized between 1882 and 1885 illustrates a landscape that is blurry, while “Still life: apples and a pot of primroses” from 1890 is clearer because it shows a closer image (**Fig. 4**,**5**) [**10**].

**Fig. 6:**
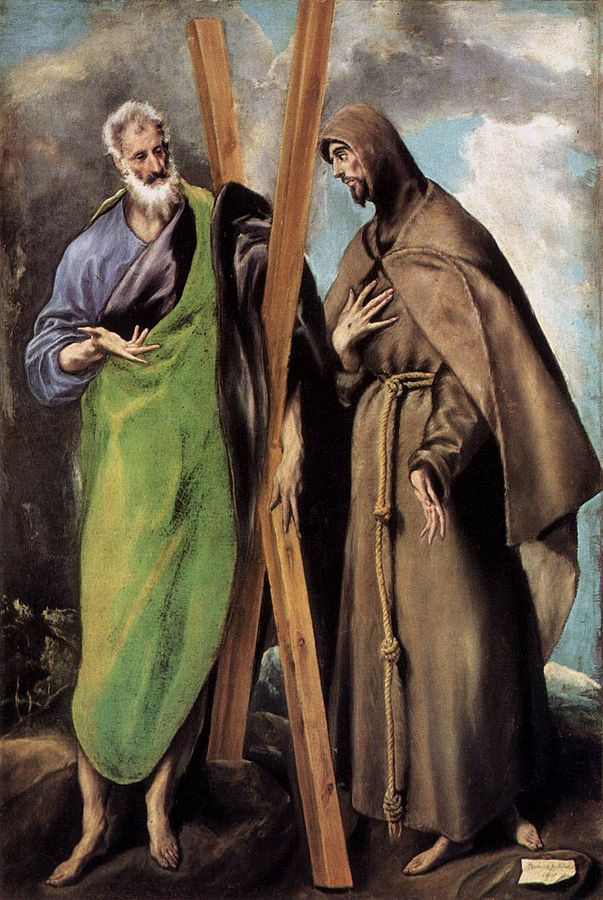
*Saint Andrew and Saint Francisc* – El Greco, 1595 [**11**]

El Greco is best known for his religious paintings and portraits, while his contributions to sculpture and architecture have unfortunately been forgotten. His paintings illustrating deformed figures can be found in churches throughout Spain. It is considered that the originality of El Greco’s paintings, with characters with elongated faces, is a consequence of the astigmatism he suffered from. These features are highlighted in the painting “Saint Andrew and Saint Francisc” from 1595 (**Fig. 6**). The subsequent, more detailed analysis of El Greco’s works, showed that this particularity of his paintings is in fact intentionally created by the artist [**11**].

*Color vision deficiency*

**Fig. 7:**
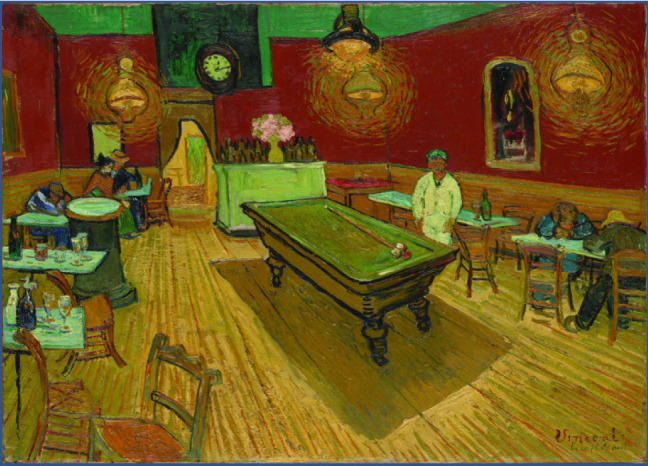
*Le café de nuit* - van Gogh, 1888 [**12**]

Vincent Willem van Gogh came from a family with a history of psychiatric pathologies, suicide, and dementia. A survey of Van Gogh’s work from 1886 to 1890 indicated that yellow-dominated paintings were numerous and episodic. One of the paintings that shows the artist’s preference for yellow is “Le café de nuit” from 1888 (**Fig. 7**). Xanthopsia appears due to digitalis ingestion or santonin overdose, but evidence of van Gogh’s use of any drug cannot be justified. The hallucinations induced by absinthe, the popular liqueur of that period, may explain certain paintings, but not the works with intense yellow colors. Excessive consumption of absinthe has, among other things, effects on the central nervous system, such as hallucinations, epileptic seizures, brain damage with risk of aggravation of mental illness and suicide attempts [**12**].

Charles Meryon was a French artist who worked in the field of etching, being affected by achromatopsia, also known as total color blindness. He is recognized as the most important engraver of France in the nineteenth century. His most famous works illustrate his gothic vision of Paris [**13**].

*Lens disorders*

Claude Monet was born on November 14th 1840 in France. He is considered one of the creators of French Impressionism. He is best known for his works that describe nature and the passage of time. At 72 years old, his vision got worse due to cataract that affected his color perception and visual acuity. The colors often looked brown-yellow, which seemed extremely frustrating to the artist, although he refused to receive surgical treatment for his condition, because he feared that his vision would worsen. Many of his paintings seem slightly blurred due to his low visual acuity. In 1923, the artist decided to have cataract surgery [**7**].

William Turner is a well-known English painter, famous for his impressive, often dramatic marine paintings. In addition to the fact that his paintings are wonderful works of art, they also highlight the evolution of the artist’s cataract [**1**].

*Ocular trauma*

**Fig. 8:**
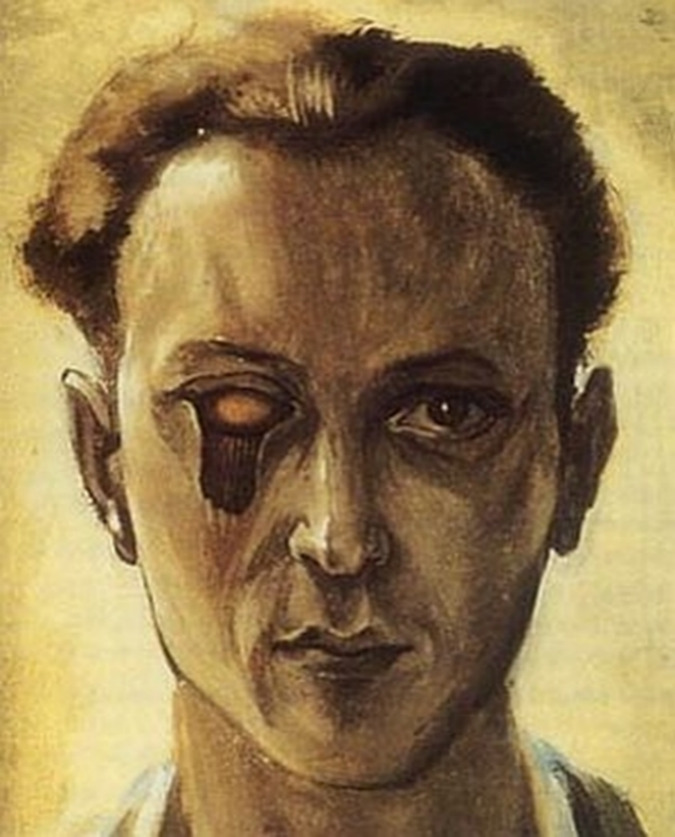
*Self-Portrait with a Plucked Eye* – Brauner, 1930

Victor Brauner was a painter, sculptor, and poet, originally from Romania. In 1930, while in Paris, he painted “Self-Portrait with a Plucked Eye”, a premonitory theme, which shows the artist’s face with his eye missing and the eye cavity hanging open (**Fig. 8**). Victor Brauner lost his left eye in 1938 during a meeting, when he had tried to settle a dispute between two friends. The trauma caused to the artist by the glass thrown by Oscar Dominguez at Esteban Francès was identical to the one painted by Brauner in 1930 [**14**].

## Conclusion

In this paper, some eye diseases that might have influenced the style of paintings were presented. Strabismus, myopia, astigmatism, retinal diseases, color vision deficiency, or cataract can influence the visual acuity of an artist. Vision can influence the techniques of artists and eye pathologies add new challenges in the art creation. Ophthalmology may not explain art, but it can help appreciate art in many ways.

**Conflict of Interest statement**

The authors state no conflict of interest.

**Acknowledgements**

None.

**Sources of Funding**

None.

**Disclosures**

None.
